# Osseointegration of implant with various surface treatments

**DOI:** 10.6026/973206300220865

**Published:** 2026-02-28

**Authors:** Ashish Kalawat, Wael Mohamed Zakaria, Gurleen Kaur, Pushkar Gupta, Swati Srivastava, Anshul Chandra, Kondaveeti Viswaja

**Affiliations:** 1 Department of Orthodontics and Dentofacial Orthopaedics, Maharaja Ganga Singh Dental Collegeand Research Centre, Sri Ganganagar, Rajasthan, India; 2 Department of Prosthodontic Dental Sciences, College of Dentistry, Qassim University, Burayadh, Qassim 51452, Saudi Arabia; 3 Intern, Kalinga Institute of Dental Sciences, KIIT Deemed to be University, Patia, Bhubaneswar-751024, Odisha, India; 4 Department of Prosthodontics and Crown & Bridge, Hitkarini Dental College and Hospital, Jabalpur, Madhya Pradesh, India; 5 Department of Periodontology and Oral Implantology, Santosh Dental College, Ghaziabad, Uttar Pradesh, India; 6 Department of Periodontology and Oral Implantology, Rama Dental College and Hospital, Kanpur, Uttar Pradesh, India; 7 Department of General Pathology, SRM Dental College, Ramapuram, Chennai-600089, Tamil Nadu, India

**Keywords:** Implant, success, surface treatment

## Abstract

Osteointegration of dental implants is significantly influenced by surface treatment. The success of dental implants is largely dependent on osseointegration. Therefore, it is of interest to examine how four frequently applied surface treatments affected implant osseointegration. Based on the surface treatment used, standardized dental implants were split into four groups. Group I received a plasma-sprayed hydroxyapatite coating; Group II received a titanium oxide nanocoating; Group III received a sandblast and acid etching; and Group IV received a UV light treatment. Following a nine-week simulated healing period under controlled settings, implants were placed in bovine bone blocks. Then they were subjected to biomechanical testing. Superior outcomes were shown by plasma-sprayed hydroxyapatite, sandblasted, acid-etched and titanium oxide nano coating group.

## Background:

Dental implants are frequently used to replace one or more lost teeth. Titanium was considered to be the preferred material for dental implants due to its exceptional biocompatibility, great corrosion resistance, high strength and low modulus of elasticity. The biocompatibility of the material being used completely determines the prognosis of dental implant treatment [1]. Dental implants typically consist of two types of titanium: a Grade 5 titanium alloy and Grade 4 titanium sometimes referred to as commercially pure titanium (CPTi) [2]. The capacity of dental implants to attain and sustain osseointegration is crucial to their success [3, 4 and 5]. An important factor in osseointegration is the implant surface. Surface roughening can improve cell attachment, bone-to-implant contact and bone cell stimulation [6]. When compared to a smooth implant surface, the roughing of the surface ensures better bone anchoring and encourages mesenchymal cell development toward an osteoblastic phenotype [7]. Enhancing the connection between the implant material and the alveolar bone is the fundamental goal of surface alterations [1]. Roughness, topography and chemical makeup of dental implants' surfaces are important factors in regulating cellular reactions and improving bone implant integration [8].

The cells engaged in anchoring are influenced by the implant surface, which has distinct osteoblastic and epithelial cell adhesion, proliferation and spreading capacities. To avoid peri-implant illness, implant surfaces must possess antibacterial properties. To reduce clinical failure, research is still needed to enhance implant material [9]. To enhance osseointegration, a variety of surface modification methods have been developed, including as chemical treatments like acid etching, mechanical treatments like sandblasting and coatings with bioactive materials like hydroxyapatite and titanium oxide nanoparticles [10, 11]. These changes are intended to improve osteoblast differentiation and bone matrix deposition, increase surface roughness and encourage protein adsorption [12, 13]. Traditional surface treatment techniques are; acid etching, sandblasting, plasma spraying, Titanium Plasma-Sprayed (TPS). Surfaces acid etching is one of the methods frequently used to treat the surface of dental implants. It involves putting an acid solution, like hydrochloric acid (HCl) or sulfuric acid (H_2_SO_4_), over the implant surface for a set period of time [1]. Another reliable technique for cleaning the surface of dental implants is sandblasting, often known as abrasive blasting. Using compressed air or a blasting equipment, aluminum oxide (Al_2_O_3_) or titanium dioxide (TiO_2_) is projected into the implant surface during this process [11]. Powdered coating materials are used during the plasma spraying process. Titanium particles that are fused to the implant surface in irregular shapes make up the roughened microstructure of titanium plasma-sprayed (TPS) surfaces [1]. Anodization, hydroxyapatite (HA) coatings, laser surface modification, nanotopography and nanocoatings are examples of advanced surface modification techniques for dental implants [14]. Therefore, it is of interest to assess the different surface modification procedure on dental implant for osseointegration and surface roughness.

## Materials and Methods:

The Department of Prosthodontics and Implantology carried out this *in vitro* investigation. Based on the surface treatment used, total 40 implants (standardized dental implants with a diameter of 4 mm and a length of 10 mm) were split into four groups with 10 samples in each group. Group I received a plasma-sprayed hydroxyapatite coating; Group II received a titanium oxide nano coating; Group III received a sandblasted and acid-etched (SLA) coating; and Group IV received a UV light treatment. Selected implants were subjected to one of the surface treatment based on group. Implants in the Sandblasted and Acid Etched Surface group underwent large grit sandblasting and acid etching using a solution of sulfuric and hydrochloric acids. Implants were coated with hydroxyapatite utilizing plasma spray technology in the Plasma Sprayed Hydroxyapatite Coating group. Implants in the titanium oxide nanocoating group were coated with titanium oxide nanoparticles via electrochemical deposition. In the UV light-treated group 10 samples were exposed to UV radiation for 15 minutes using UV activation equipment. The UV light was delivered as a spectrum combination using a single source (λ = 360 nm and λ = 250 nm). The human jawbone was replicated using 20 x 20 x 10 mm bovine cortical bone blocks. Before being used, bone blocks were disinfected and kept at 4°C in phosphate buffered saline. Each implant was placed under regulated conditions into a predrilled hole in the bone blocks. To replicate the physiological milieu during osseointegration, the bone blocks containing implants were incubated in simulated body fluid (SBF) at 37°C for eight weeks. The test samples' surface characteristics were evaluated using a scanning electron microscope (SEM) following surface treatment. A 3000x magnification was used to take all of the sample photos. A digital optical profilometer with a stylus speed of 0.5 mm/s was linked to computer software. This measure the surface roughness after the surfaces of all the groups had been treated. The surface-treated implants were then assessed for pull-out strength, surface roughness and Bone Implant Contact (BIC) percentage. After the bone blocks were sectioned, histomorphometric analysis was done to determine the percentage of bone Implant Contact (BIC). A light microscope was used to take pictures and ImageJ software was used to determine the BIC percentage. A universal testing machine was used to do mechanical testing for Pull out Strength. Each implant's dislodging force from the bone block was measured in Newtons (N). SPSS statistical software version 24.0 (IBM), one-way ANOVA and Tukey's post hoc test were used to statistically analyze the collected data. Statistical significance was defined as a P value of less than 0.05.

## Results:

The current study shows that surface treatments have a major impact on dental implant osseointegration. The highest bone implant contact (Table 1) and pull out strength (Table 2) were found in the Titanium Oxide Nano Coating treatment, which was followed by the Plasma Sprayed Hydroxyapatite Coating, SLA and UV light group. Titanium Oxide Nano Coating treatment had the highest surface roughness followed by Plasma Sprayed Hydroxyapatite Coating , SLA and UV light group (Table 3).

## Discussion:

The current research shows that surface treatments have a major impact on dental implant osseointegration. The best performance was achieved by the Titanium Oxide Nano Coating treatment, which was followed by the Plasma Sprayed Hydroxyapatite Coating, SLA and UV light group. These results are consistent with earlier research highlighting the crucial role that implant surface modifications play in improving bone-implant interaction [3, 8]. Surface treatment is used in dental implants to change the surface energy and topography. This enhances wettability, encourages cell growth and proliferation and accelerates the osseointegration process [15]. The surface roughness of treated implants on SEM photos has been measured using a variety of techniques. Numerous studies have shown that the osseointegration process and peri-implant bone healing can be influenced by the bone-implant interface [2].

The effects of four popular surface treatments on implants were evaluated by Yadav *et al.* They came to the conclusion that surface treatments had a major impact on dental implant osseointegration. Better outcomes were shown by hydroxyapatite coatings sprayed with plasma and titanium oxide nanocoating [16]. Marenzi *et al.* examined the microstructural conformation and chemical makeup of dental implants that had undergone various surface treatments. They came to the conclusion that the acid-etched surfaces had greater peaks and deeper troughs than the sandblasted ones. Because of this, the stress generated by the peri-implant bone during surgical implant insertion can more readily damage acid-etched surfaces [2]. Anka *et al.* assessed the effectiveness of three methods for decontaminating implants. They came to the conclusion that the surface roughness of SLA or DAE implants was not considerably changed by any of the three cleaning methods [17]. Jalaluddin evaluated how different surface treatments affected the surface of the implant. In comparison to the UV light and control groups, they found that the SLA-treated group exhibited the highest surface roughness [18]. Increased implant-bone osseointegration has been observed in dental implants after surface treatment. Compared to implants with SAE areas, those with hydrophilic surfaces osseointegrated faster [19]. According to Karni *et al.* surfaces that were acid-etched showed better osseointegration, indicating that surface modification can improve implant success [20]. From the present study we found improved surface roughness and improved osseointegration of implant. Further researches are required to confirm the findings.

## Conclusion:

Surface roughness, chemical composition and bioactivity all work together to determine implant performance, as evidenced by the notable group disparities.

## Advancement to knowledge:

Bioactive osseointegration, in which surface treatments actively encourage the host bone to renew and connect directly to the implant. This has replaced basic mechanical retention in dental implantology. Modern surfaces optimize surface chemistry, wettability and nanotopography to speed up this process and cut healing durations from months to weeks.

## Figures and Tables

**Table 1:** Percentage of bone implant contact (BIC)

**Groups**	**Surface modification type procedure**	**BIC (%)**
Group I	Plasma-Sprayed Hydroxyapatite Coating	78.46±4.3
Group II	Titanium Oxide Nano-Coating	82.7±3.7
Group III	Sandblasted and Acid-Etched (SLA)	62.4±3.2
Group IV	UV light-treated group	47.4±4.6

**Table 2:** Pull out strength (N)

**Groups**	**Surface modification type**	**Pull-Out Strength (N)**
Group I	Plasma-Sprayed Hydroxyapatite Coating	271.38±10.6
Group II	Titanium Oxide Nano-Coating	316.3±13.3
Group III	Sandblasted and Acid-Etched	214.6±11.4
Group IV	UV light-treated group	161.4±9.3

**Table 3:** Assessment of surface roughness on the implant surface

**Groups**	**Surface modification type**	**surface roughness (nean± SD)**
Group I	Plasma-Sprayed Hydroxyapatite Coating	0.689±0.05
Group II	Titanium Oxide Nano-Coating	0.727±0.03
Group III	Sandblasted and Acid-Etched	0.674±0.12
Group IV	UV light-treated group	0.671±0.04
